# Draft Genome Sequence of Two Strains of *Xanthomonas arboricola* Isolated from *Prunus persica* Which Are Dissimilar to Strains That Cause Bacterial Spot Disease on *Prunus* spp.

**DOI:** 10.1128/genomeA.00974-16

**Published:** 2016-09-08

**Authors:** Jerson Garita-Cambronero, Ana Palacio-Bielsa, María M. López, Jaime Cubero

**Affiliations:** a Departamento de Protección Vegetal, Instituto Nacional de Investigación y Tecnología Agraria, Madrid, Spain; b Centro de Investigación y Tecnología Agroalimentaria de Aragón, Instituto Agroalimentario de Aragón-IA2 (CITA-Universidad de Zaragoza), Zaragoza, Spain; c Instituto Valenciano de Investigaciones Agrarias, Valencia, Spain

## Abstract

The draft genome sequences of two strains of *Xanthomonas arboricola*, isolated from asymptomatic peach trees in Spain, are reported here. These strains are avirulent and do not belong to the same phylogroup as *X. arboricola* pv. pruni, a causal agent of bacterial spot disease of stone fruits and almonds.

## GENOME ANNOUNCEMENT

*Xanthomonas arboricola* is a Gram-negative species that comprises nine subspecific phylogroups (1, 2). In addition, some strains that differ from the pathovars *X. arboricola* pv. pruni and *X. arboricola* pv. juglandis, isolated from the plant genera *Prunus* and *Juglans*, have also been described. These strains are unable to cause disease because they lack some essential characteristics related to pathogenesis, such as the type III secretion system and related effectors (3, 4).

The two new genomes presented here, which belong to the avirulent strains CITA 14 and CITA 124, were compared to the genomes of other *X. arboricola* strains (3–9) and could be helpful in understanding the pathogenesis and evolution in this species. Moreover, the genomes will be useful for generating accurate diagnostic tools to differentiate the quarantine pathogen *X. arboricola* pv. pruni from other nontarget strains, thereby avoiding unnecessary control measures that could result in high economic losses as occurred with other xanthomonads (10, 11).

Strains CITA 14 and CITA 124 were isolated from asymptomatic peach trees (*Prunus persica*) in Spain and were deposited in the Centro de Investigación y Tecnología Agroalimentaria de Aragón (CITA) in Zaragoza, Spain. To confirm their identification, a multilocus sequence analysis using partial sequences of the housekeeping genes *dnaK*, *fyuA*, *gyrB*, and *rpoD* was performed (12). By means of such analysis, these strains were not clustered into any of the established pathovars of *X. arboricola*. CITA 14 and CITA 124 were enclosed in a group with CITA 44, another atypical strain of *X. arboricola* isolated from the rootstock Santa Lucía SL-64 (*Prunus mahaleb*) (4), which shows sequence identities of 98.3% with strain CITA 14 and 98.9% with strain CITA 124.

Genome sequencing was performed using Ion Torrent sequencing technology (PGM, Life Technologies) at STAB VIDA, Caparica, Portugal (733.06 Mb, 100-fold coverage for CITA 14 and 403.99 Mb, 50-fold coverage for CITA 124). *De novo* assembly was conducted using CLC Genomics Workbench version 8.5.1 (CLC bio, Denmark) and MIRA version 4.0 (13), and the subsequent merging of the two assemblies was done using Sequencher version 5.4 (Gene Codes Corporation, USA). Total lengths of 4,746,212 bp (65.60% G+C content) and 4,752,241 bp (65.8% G+C content) were generated and placed in 72 (*N*_50_, 108,424 bp; maximum contig length, 265,354 bp) and 128 contigs (*N*_50_, 60,818 bp; maximum contig length 189,085) for CITA 14 and CITA 124, respectively.

Annotations were conducted using the NCBI Prokaryotic Genome Annotation Pipeline version 3.1 (14) and the Rapid Annotations using Subsystem Technology server (15). The annotation for strain CITA 14 detected 3,974 coding sequences, four rRNAs, and 53 tRNAs, representing 439 subsystems; meanwhile, for strain CITA 124, 3,797 coding sequences, three rRNAs, and 50 tRNAs, representing 442 subsystems, were detected. A detailed genome comparative analysis will be addressed in a future publication.

### Accession number(s).

These whole-genome shotgun projects have been deposited in DDBJ/EMBL/GenBank under the accession numbers LXIB00000000 for strain CITA 14 and LXKK00000000 for strain CITA 124. The versions described in this paper are the first versions, LXIB01000000 and LXKK01000000.
